# Rose rosette emaravirus dynamics in eriophyoid mites: implications for virus transmission

**DOI:** 10.1007/s10493-026-01135-w

**Published:** 2026-04-22

**Authors:** Tobiasz Z. Druciarek, Alejandro J. Rojas, Ioannis E. Tzanetakis

**Affiliations:** 1 Department of Entomology and Plant Pathology, Division of Agriculture, University of Arkansas, Fayetteville, AR USA; 2 Department of Plant Protection, Warsaw University of Life Sciences, Warsaw, Poland; 3 Department of Plant, Soil and Microbial Sciences, Michigan State University, East Lansing, MI USA

**Keywords:** *Emaravirus rosae*, *Phyllocoptes fructiphilus*, *Phyllocoptes adalius*, Transmission, Virus titration

## Abstract

Understanding the interaction between rose rosette emaravirus (RRV) and its vectors is pivotal in addressing the epidemic outbreak of rose rosette disease. This study employed quantitative real-time RT-PCR to assess RRV concentration in *Phyllocoptes fructiphilus* and *P. adalius*, providing insights into the viral dynamics and vector competency. Our findings suggest an increase in virus titer within *P. fructiphilus*, a confirmed vector species, unlike *P. adalius*, highlighting its non-vector status. Furthermore, the study highlights the variability in virus concentration in mites over time, underlining possible developmental stage-specific response and influence of mite lifestyle on RRV retention and replication. This research is the first step in understanding the virus-mite interaction mechanisms, which is essential for developing effective management strategies against rose rosette and potentially other emaravirus-caused diseases.

**Supplementary Information:**

The online version contains supplementary material available at 10.1007/s10493-026-01135-w.

## Introduction

Eriophyoid mites (phylum Arthropoda; class Arachnida) are the smallest arthropod virus vectors and cause significant losses in food, tree and ornamental crops across the globe (Büttner et al. 2023; Rehanek et al. 2022; Druciarek and Tzanetakis 2025). Approximately 5000 species have been described, but the actual number is hypothesized to be significantly higher (Stenger et al. 2016; Xue et al. 2023). As of 2026, eriophyoid mites are verified or suspected vectors of ~ 60 plant viruses (Digiaro et al. 2024; Druciarek et al. 2024); however, in the metagenomics era, the rate of identifying vectors is not keeping up with the increasing number of virus discoveries (Villamor et al. 2019; Maclot et al. 2020). There is an even greater knowledge gap in understanding the dissemination mechanisms of eriophyoid-transmitted viruses (Druciarek et al. 2019; Druciarek and Tzanetakis 2025).

The emerging genus *Emaravirus* (family *Fimoviridae*; order *Elliovirales*) comprises of over 40 classified and putative species of eriophyoid-transmitted viruses with worldwide distribution and economic impact (Digiaro et al. 2024; Rehanek et al. 2022). The multi-segmented negative-sense, single-stranded RNA emaraviruses encode four core proteins; replicase, glycoprotein, nucleoprotein and movement protein and several accessory proteins that are involved in RNA interference, proteolysis and potentially other functions (Rehanek et al. 2022).

The heptasegmented rose rosette emaravirus, RRV (species: *Emaravirus rosae*) is considered one of the most economically significant emaraviruses, as infected plants die within two to five years after the onset of symptoms (Di Bello et al. 2018), affecting the profitability and sustainability of commercial operations and landscapers in the United States (Druciarek et al. 2023; Windham et al. 2023).

RRV is vectored by *Phyllocoptes fructiphilus* Keifer (Di Bello et al. 2018) and the recently identified *P. arcani* Druciarek, Lewandowski and Tzanetakis (Druciarek et al. 2021, 2023). It remains unclear whether the virions are transiently and reversibly retained or if they circulate and replicate within the mite’s body. To address this, we tested the hypothesis of RRV replication in the mite body by assessing genome numbers in the better-studied vector *P. fructiphilus* and non-vector species *P. adalius*. This research provides a deeper understanding of interactions between RRV and mites and offers new perspectives on factors influencing the dissemination of emaraviruses.

## Materials and methods

### Maintenance of mites and plants

Non viruliferous *P. adalius* and *P. fructiphilus* colonies used in the study of Druciarek et al. (2023) were maintained on potted KnockOut^®^ roses (*Rosa* × *hybrida* ‘Radrazz’) and tested as described in Di Bello et al. (2018). *P. adalius* was included in the study because it is the only eriophyoid mite infesting roses that is a verified non-vector of the virus, even though it does not share the same lifestyle as *P. fructiphilus* (Druciarek et al. 2023; Keifer 1939, 1940). RRV was maintained on infected KnockOut^®^ roses by *P. fructiphilus-*mediated transmission. The presence of the virus was verified by amplifying a 271 and a 201 base fragment of RNA 3 (suppl. Material, suppl. Table 1) which were Sanger-sequenced and matched virus isolates available in NCBI. Plants with mite colonies and RRV-source plants were maintained in separate environmental growth chambers (14 h light:10 h dark, 20 °C, 70% RH) and monitored for several months before being used in experiments.

### Construction of standard curves for quantitative analysis

Standard curves were generated for each target to determine the number of RRV genome copies in mites. The emaravirus-specific primer PDA213 (Di Bello et al. 2015) was used for reverse transcription (RT), generating cDNA from viruliferous *P. fructiphilus* specimens as described below. An amplicon encompassing the virus RNA 3 was generated, whereas, for an internal control/reference gene, an amplicon targeting the 18 S rDNA region of the mite was also obtained directly from genomic mite DNA (suppl. material). Initially, three additional reference gene candidates (actin, polyubiquitin, and GAPHD) were evaluated for stability under experimental conditions (data not shown). The 18 S rDNA consistently demonstrated the lowest variation in the threshold cycle (C_t_) values across all samples in both mite species, indicating its robustness and reliability as a reference gene for normalization in this study. DNA concentrations of sequenced amplicons were determined with a Qubit 3.0 fluorometer (Life Technologies), and the copy number of each target was calculated using the formula:

$${V_c}=({C_a} \times {N_A})/({l_a} \times {m_b})$$

where *V*_*c*_ is the virus copies/µL, *C*_*a*_ the amplicon concentration in ng, *N*_*A*_ the Avogadro’s constant (6.02 × 10^23^), *l*_*a*_ the amplicon length in base pairs and *m*_*b*_ the molecular mass of 1 bp in ng/mol (660 × 10^9^). Ten-fold dilutions (10^6^−10^2^ copies) were prepared, and qPCR was performed with two technical replicates, as described below (suppl. Fig. 1). Curves were built by plotting C_t_ values versus the log_10_ of the target copy number. The amplification efficiency (E) of each assay was calculated using the equation *E* = 10^(− 1/S)^, where *S* is the slope of the corresponding curve.

### Quantification of virus titer

Quantification of viral and reference gene copies was performed using a modified version of the direct RT-PCR method described by Druciarek et al. (2019; Fig. 2 assay) with standards and cDNAs from mite and plant samples assayed by Taqman^®^ qPCR (suppl. Fig. 1, supplemental material). Individual eriophyoids were crushed with a metal pin (size 000) in 5 µL of TE buffer. Reverse transcription was then carried out in a 20 µL reaction containing 4 µL 5 × RT buffer, 100 U Maxima Reverse Transcriptase (Thermo Fisher Scientific, catalog no. EP0741), 0.4 mM dNTPs (Invitrogen, catalog no. 18427088), 300 ng random primers (Invitrogen, catalog no. 48190-011), 10 U RiboLock RNase Inhibitor (Thermo Fisher Scientific, catalog no. EO0381), and water to volume. The sample was incubated at 50 °C for 1 h followed by 10 min at 75 °C. Five (5) µl cDNA was used in the qPCR reaction. Samples were analyzed in two technical replicates for RRV RNA3 and mite 18 S rDNA. Each qPCR plate included cDNA from symptomatic RRV-infected rose tissue (Knock Out^®^ ‘Radrazz’) as a positive control, and cDNA from RRV-free rose tissue plus a no-template control as negatives with total nucleic acids extracted and reverse transcribed as described in Poudel et al. (2013). C_t_ values from RRV-containing samples were compared with the standard curves to determine absolute quantities of the targets with amounts normalized by quantities of the corresponding reference gene.

### Virus titer through time

**Fig. 1:**
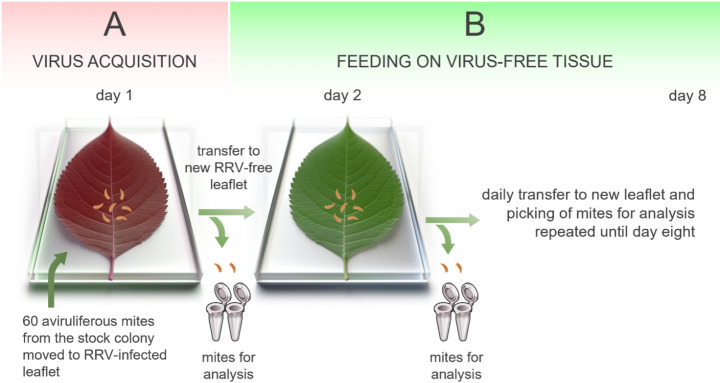
Schematic representation of rose rosette emaravirus (RRV) quantification assay. (**A**) Virus acquisition by immature mites moved to RRV-infected material and fed for 24 h. (**B**) Daily transfer of developing mites to new, RRV-free tissue with two mites taken daily for analysis. The artwork was partially produced using the Midjourney bot via a Discord server at https://discord.com/invite/midjourney

Immature mites (larvae) from each non-viruliferous colony were transferred to modified Munger cells (60/cell) (Druciarek et al. 2014; suppl. Fig. 2) containing detached, RRV-infected leaflet and kept for 24 h in cells placed in an environmental growth chamber (14 h light :10 h dark, 27 °C, 63% RH) for virus acquisition. The 24 h acquisition access period was selected based on previous studies on *P. fructiphilus* and *P. adalius* biology and RRV transmission attributes (Druciarek et al. 2014; Di Bello et al. 2018). There were eight cells for *P. adalius* and 12 for *P. fructiphilus.* On the second day, two mites from each cell (16 *P. adalius* and 24 *P. fructiphilus/* day) were collected in separate tubes containing TE buffer and stored at −80 °C for subsequent analysis. The rest of the mites were moved to a new cell with a detached, RRV-free leaflet for 24 h. This process of collecting two individuals and transferring the remaining mites to a new cell with a detached RRV-free leaflet continued daily until day 8 (Fig. 1). Additionally, 16 and 24 individuals, for *P. adalius* and *P. fructiphilus* respectively, were gathered from the nonviruliferous mite stock colonies just before their initial transfer to the RRV-infected leaflets for virus acquisition. In total 144 *P. adalius* and 216 *P. fructiphilus* individuals were collected and analyzed throughout the experiment.

## Statistical analyses

The resulting qPCR runs were extracted using batch processing mode in CFX Maestro v2.3 (Bio-Rad, Hercules, CA) and imported into R version 4.2.1 (R Core Team, Vienna, Austria). Since there are multiple independent qPCR runs, tenfold standards (10^6^−10^2^ copies) were included on every plate for RRV and mite rDNA. The data was analyzed to determine whether there were differences between plates before combining the data for further analysis. A linear model was employed, using C_t_ values as the response variable and log-transformed copies as a factor while treating the plate as a random factor. This approach was used to assess variability across plates before merging the results for comprehensive analysis. For the merged data, an infection coefficient (IC) was calculated as follows:

$$\mathrm{IC} = \mathrm{RRV}/\mathrm{mite}\ \mathrm{rDNA}\ \mathrm{concentration}$$

An additional approach to assess infection efficacy was to use a normalized Infection Coefficient (nIC), defined as dividing the C_t_ value of the vector by the C_t_ value for the cDNA of RRV (nIC = C_t_ mite/C_t_ RRV).

Linear regression analysis was performed to assess the virus concentration in each mite species. Total DNA was quantified from the host mites via qPCR, and the results were compared with the corresponding virus concentrations estimated via RT‒qPCR. A constant was added to all virus samples to adjust for zero values, and DNA concentrations for viruses and mites were log_10_-transformed. A Pearson correlation was calculated to determine whether there was a significant correlation between the two variables. To investigate the differences in the infection coefficient or virus concentration across the eight feeding events (days), the infection coefficient was analyzed over eight days (events). A repeated measures analysis was performed to identify any differences across these events. Both the mite species and the acquisition events were treated as factors in a two-way ANOVA for repeated measures. Significant effects were further evaluated using post-hoc tests, specifically pairwise comparisons with adjustments using the Bonferroni method for multiple comparisons. All analyses were conducted using R version 4.2.

## Results

The influence of independent PCR plates was included as a random factor in the analysis, explaining only 0.013 and 0.015 of the variances in the virus and the mite rDNA concentration, respectively. Additionally, the homogeneity of regression slopes across both assays was tested and was statistically insignificant (RRV *p* = 0.328, mite rDNA *p* = 0.808) (suppl. Figure 1).

**Fig. 2:**
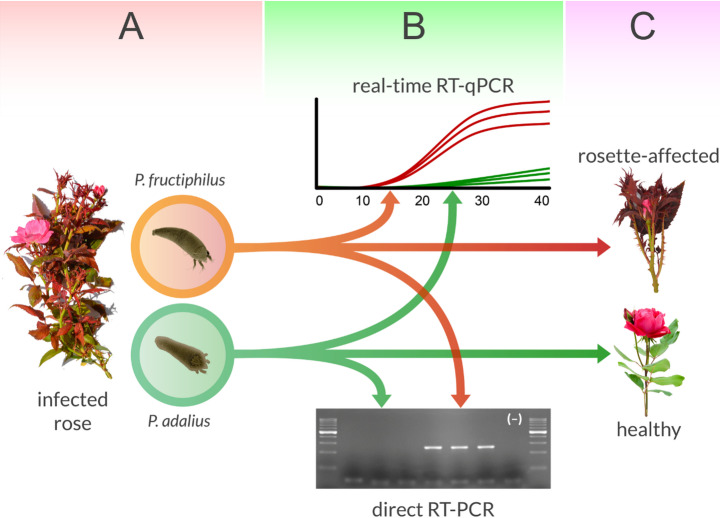
Schematic representation of rose rosette emaravirus (RRV) transmission competency by eriophyoid mites. **(A)** RRV is acquired by both *Phyllocoptes* species feeding on infected rose. **(B)**However, only *P. fructiphilus* has enough of a virus load to obtain a positive amplicon in semi-quantitative RT-PCR in 20 and 30 cycles unlike *P. adalius* (Druciarek et al. 2019), and RT-qPCR assay suggests replication in this species. **(C)** Transfer of viruliferous mites to recipient plants results in successful transmission and development of symptoms only in the case of *P. fructiphilus* (Druciarek et al. 2023)

Analysis of the change in virus concentration in relation to the rDNA concentration of each of the two mite species revealed that for RRV - *P. adalius* rDNA concentration had a statistically insignificant regression (R² = 0.045, *p* = 0.29), suggesting that the virus did not replicate in the mites (Fig. 2). In contrast, a positive correlation was observed between RRV and *P. fructiphilus* rDNA (R² = 0.36, *p* = 2.2e^− 16^; Fig. 2), indicating that the virus concentration increases.

**Fig. 3:**
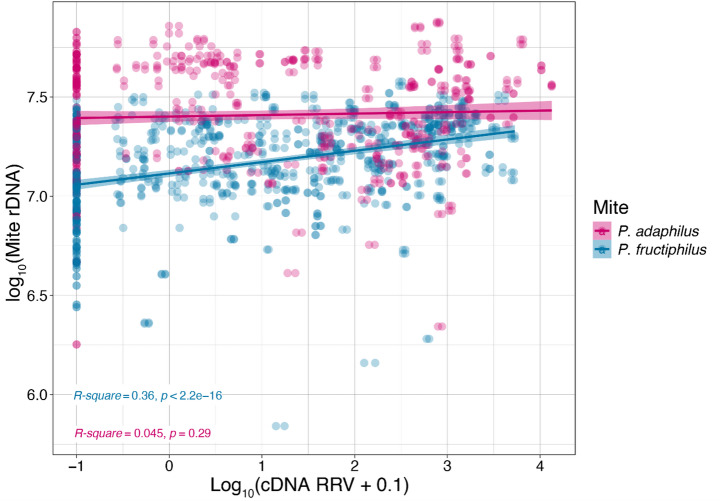
Correlation of virus load (log_10_ of cDNA ng/µL) and the corresponding host mite DNA (log_10_ of mite rDNA). Bands represent a 95% confidence interval of the fit line. Correlations were evaluated using Pearson correlation, and R-square and p-value for both *Phyllocoptes adalius* and *P. fructiphilus* were included

The normalized infection coefficient showed that both species acquire RRV (Fig. 3). The overall infection coefficient varied between 0.3 and 0.6, with *P. adalius* displaying higher variability. Most feeding events yielded similar results; however, on day 5, the infection coefficient for *P. fructiphilus* surpassed that of *P. adalius*.

**Fig. 4:**
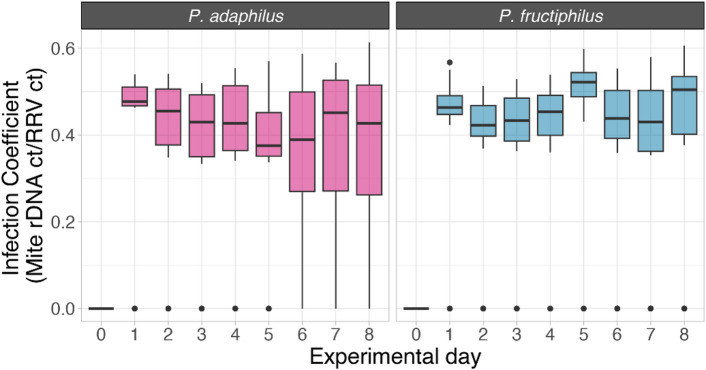
Box plot of normalized infection coefficient of rose rosette emaravirus to *Phyllocoptes adalius* and *P. fructiphilus* across experimental days. Day 0 represents mites before acquisition, and day 1 represents mites collected immediately after the 24 h acquisition access period. Dots represent outliers

Repeated measures analysis of these fluctuations indicated significant differences in the infection coefficient at days 0, 1, 2, 5, and 8 (Fig. 4). In particular, *P. adalius* had higher coefficients on days 1 and 2, though with less significance compared to the instances where *P. fructiphilus* dominated (days 0, 5, and 8). While the trend was consistent in the initial events, day 5 marked a notable increase (*p* = 1.35e^− 14^) in virus concentration for *P. fructiphilus*.

**Fig. 5:**
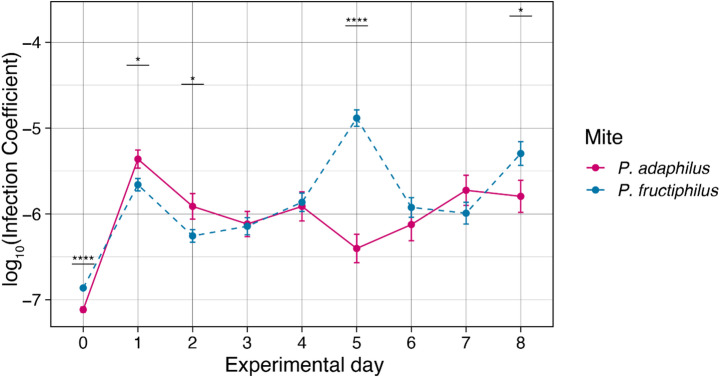
Experimental day dynamics of rose rosette emaravirus (RRV) log_10_ infection coefficient derived from the cDNA RRV divided by the mite rDNA. Day 0 represents mites before acquisition, and day 1 represents mites collected immediately after the 24 h acquisition access period. Points represent the mean of 16 and 24 individual mites for *Phyllocoptes adalius* and *P. fructiphilus*, respectively, and error bars represent standard error. Significant differences per event were calculated with a pairwise test, and p-values adjusted with Bonferroni. (Significance levels: *=0.05, **=0.01, ***=0.001, ****=0.0001)

Variability in the infection coefficient, especially the spike observed in *P. fructiphilus* on day five, suggests factors influencing RRV dynamics at different developmental stages of the mites (Fig. 5). Notably, day five also coincided with RRV transmission in the study by Di Bello et al. (2018).

## Discussion

Our study advances the understanding of virus dynamics by quantitatively monitoring virus concentrations in mites over time after transient exposure to RRV-infected tissue. To ensure accurate measurements and confirm that the obtained values reflect virus presence within the mites’ tissues, we cleared the digestive tract by transferring them to virus-free arenas after 24 h of exposure, preventing further uptake of infected plant material. The mites were then transferred daily to fresh virus-free tissues, and viral concentrations were quantified over seven consecutive days. The use of *P. adalius*, a non-vector species, alongside *P. fructiphilus*, a confirmed RRV vector, provided a new perspective on vector competency and virus-mite interaction dynamics (Fig. 2; Di Bello et al. 2018; Druciarek et al. 2019, 2023). Notably, this is the first study to perform quantitative monitoring of virus concentrations in individual eriophyoid mites. While this study provides a foundational framework, further investigation is needed to address the complexities of virus titer changes and replication dynamics.

Our group has evaluated alternative approaches that could be employed to investigate changes in virus titer and replication in mites. One such approach involved detecting the positive strands of the virus, but results were inconclusive (P. Di Bello and I.E. Tzanetakis, unpublished), likely due to the co-packaging of complementary virus strands within virions, a phenomenon observed in other bunyaviruses (Schreur et al. 2018) including the European mountain ash ringspot-associated emaravirus (EMARaV), the type member of the genus (Mielke-Ehret et al. 2010). We also considered the use of fluorescent immunology to detect particles in the mite bodies; however, antibodies raised against the nucleoprotein lacked sufficient sensitivity, failing to reliably detect the virus even in plant tissue where it was consistently detectable after 20 PCR cycles (P. Di Bello and I.E. Tzanetakis, unpublished). Another approach considered was sequencing small RNAs from mites. However, this method is highly inefficient for negative-strand RNA viruses, particularly RRV, as we often failed to detect virus small RNAs extracted from one gram of tissue (T. Ho and I.E. Tzanetakis, unpublished), let alone from a single eriophyoid mite.

For those reasons we opted for quantitative RT-PCR, which enabled reliable RRV and mite rDNA assessment in *P. fructiphilus* and *P. adalius* (Figs. 3 and 4). The infection coefficient, derived from RRV/rDNA concentrations and C_t_ value ratios, revealed new aspects of RRV dynamics. Notably, there was a positive correlation between the virus concentration and the vector rDNA concentration in *P. fructiphilus*; as the number of rDNA copies increased (presumably as immature mites develop into adults), so did the virus concentration within the mite, indicating active replication of RRV in the confirmed vector. These findings align with the results of Druciarek et al. (2019), where end-point PCR amplicons (30 cycles) were obtained only from *P. fructiphilus* and not *P. adalius* individuals.

Considering developmental timelines reported for both species (Druciarek et al. 2014; Kassar and Amrine 1990), it is highly probable that the mites had reached an adult stage by this point.

We initiated the study with cohorts of immatures (larvae) to ensure virus acquisition and sufficient survival rates throughout the experiment. However, our methodology, involving 24-hour sampling intervals, did not allow for precise identification of the specific life stages during sampling. A more detailed insight into the developmental stage-specific virus responses is needed, similar to studies conducted on aphids and thrips (Li et al. 2020; Schneweis et al. 2017).

The variability observed during the first two days (Fig. 5) may be attributed to the differing lifestyles of studied eriophyoid species (Sabelis and Bruin 1996). As noted, *P. adalius* was selected because it is the only verified non-vector of RRV. Its vagrant lifestyle is well-suited to flat leaf surfaces in the experimental arena (Druciarek et al. 2014). In contrast, a refuge-seeking lifestyle of *P. fructiphilus*, which involves hiding in areas like flower buds and petiole bases, may result in less frequent feeding on the arena’s exposed surface, as these mites spend significantly more time searching for suitable shelter (Keifer 1939, 1940; T. Druciarek, personal observation). Despite these differences, both mite species demonstrated the ability to carry RRV for over a week. The higher variability observed in *P. adalius* might indicate different mechanisms of RRV retention.

Comparisons can be drawn with other plant-infecting members of the *Bunyavirales*, especially orthotospoviruses (family*Tospoviridae*). It has been shown that in tomato spotted wilt orthotospovirus (TSWV), the best studied member of the group, transmission dynamics differ significantly between vector species (Nagata et al. 2002). TSWV vector competence is influenced by virus replication in larvae and subsequent migration to salivary glands. A key question for future research is whether emaraviruses, similar to orthotospoviruses, require acquisition during specific developmental stages – larval, nymphal or adult – for successful transmission (Mou et al. 2021), and whether vector competence changes as mites develop (Rotenberg et al. 2015; Wetering et al. 1996).

Emaraviruses and orthotospoviruses are characterized by similar genome structures and virion architecture, leading researchers to suggest that emaraviruses might be transmitted in a persistent, propagative manner, as observed in orthotospoviruses (Chen et al. 2019). While some studies support a persistent, propagative mode (Di Bello et al. 2018; Martelli et al. 2013), others suggest a semipersistent mode (Kulkarni et al. 2002). Our study provides evidence of RRV replication in *P. fructiphilus*; however, it is possible that transmission attributes vary across different emaravirus/vector/host pathosystems.

Our understanding of the virus-mite interactome is at a nascent phase. A significant knowledge gap exists on intricate transmission mechanisms and molecular determinants of virus dissemination in mites (Druciarek et al. 2019; de Lillo et al. 2021). Bridging these gaps is crucial for devising innovative, selective, and durable control measures, as has been achieved with other viral systems (Montero-Astúa et al. 2014; Patterson et al. 2020; Tabein et al. 2020; Zhou and Tzanetakis 2020). The frequency and scale of outbreaks caused by arthropod-borne diseases, such as rose rosette, are increasing due to factors associated with climate change, human demographics, and globalization (Jones and Naidu 2019; Singh et al. 2023). By quantifying virus concentrations in individual mites, our methodology offers new insight to the understanding of eriophyoid-borne diseases. This approach is versatile, suitable for further investigations, and applicable to other pathosystems. It represents a significant step toward understanding virus dynamics in mites and provides a foundation for developing practical tools to mitigate threats to agriculture and biodiversity.

## Supplementary Information

Below is the link to the electronic supplementary material.

Supplementary material 1 (DOCX 722.2 kb)

## Data Availability

TZD has generated the data which have been curated by AJR and IET. TZD and IET conceived, designed and conducted experiments. AJR, TZD and IET analyzed the data. All participated in writing the paper and internal review. All authors have read and approved the final manuscript.

## Declarations

### **Declarations of generative AI and AI-assisted technologies in the writing process**.

During the preparation of this manuscript, the authors used ChatGPT to assist with language refinement. After using this tool, the authors thoroughly reviewed and edited the content and take full responsibility for the final version of the publication.
