# Supplementary material for: Rose rosette emaravirus dynamics in eriophyoid mites: implications for virus transmission
Source: Exp Appl Acarol. 2026 Apr 22;96(4):45. doi: 10.1007/s10493-026-01135-w (PMC13102888; doi:10.1007/s10493-026-01135-w)
Supplement: Supplementary file 1 — Supplementary material 1 (DOCX 722.2 kb) [file 10493_2026_1135_MOESM1_ESM.docx]

**Supplementary Information**

Supplementary material file: Primers and probes used in the experiments, sequences of targeted regions, details on quantification of RRV titer using direct RT-qPCR and TaqMan assay, and qPCR standard curves generated for multiple independent runs.

**Supplementary table 1.** Primers and probes used for TaqMan-based RT-qPCR detection of rose rosette emaravirus, mite reference gene and generation of standard curves.

| Gene | Primer or  probe name | Sequences (5’-3’) | Dye | Amplicon size | Reference |
| --- | --- | --- | --- | --- | --- |
| *TaqMan® RT-qPCR detection* | | |  |  |  |
| RNA3 RRV | RRV2F | TGCTATAAGTCTCATTGGAAGAGAAA | - | 104 | Dobhal et al., 2016 |
|  | RRV2R | CCTATAGCTTCATCATTCCTCTTTG | - |  |  |
|  | RRV probe-2 | TGCTAGAGA/ZEN/CATTGGTACAACAAGCAA/3IABkFG/ | FAM |  |  |
| 18S rDNA | 18S-TqM-F2 | TGGGTTGCGATTCCTTTAAT | - | 107 | this study |
|  | 18S-TqM-R2 | AATCATACTTCCCCCGGAAC | - |  |  |
|  | 18S-TqM-P2 | CTCCGATCA/ZEN/TTATGATCCACCCAGC/3IABkFQ/ | FAM |  |  |
| *Generation of standard curves* | | |  |  |  |
| RNA3 RRV | RNA3flanF1 | CGTATTCACAAGCTAGAGACTACTCC | - | 201 | this study |
|  | RNA3flanR2 | ATTGTGCACCTCTATCAGCAGCT | - |  |  |
| 18S rDNA | Flan18S-TqM-F2 | GATCAGATACCGCCCTAGTTC | - | 189 | this study |
|  | Flan18S-TqM-R2 | CCCTTCCGTCAATTCCTTTAAG | - |  |  |

**An amplicon encompassing the virus (RNA3) target region:**

CGTATTCACAAGCTAGAGACTACTCCTTTCGATGATGCTATAAGTCTCATTGGAAGAGAAAACATATCTGAAGCATATGTTGAACTTGCTAGAGACATTGGTACAACAAGCAAATCAAAGAGGAATGATGAAGCTATAGGCAAGTTCAAAGAACTGATCAAGAACTTTGCTCCTGCTTTAGCTGCTGATAGAGGTGCACAAT

**An amplicon encompassing the mite rDNA (18S) target region:**

GATCAGATACCGCCCTAGTTCTAACCATAAACGTTGCCAACTAGCAATTGGGTTGCGATTCCTTTAATCGGAGTGTAAAAACTCCGATCATTATGATCCACCCAGCGGCTCTCGTAGGGAAACCAAAGTGTTTGGGTTCCGGGGGAAGTATGATTGCAAAGTTGAAACTTAAAGGAATTGACGGAAGGG

**Quantification of RRV titer using direct RT-qPCR and TaqMan assay**

Individual eriophyoids were crushed with a metal pin (size 000) in 5 μL of TE buffer and combined with an RT mix containing 4 μL 5 × RT buffer and 100 U Maxima Reverse Transcriptase (Thermo Fisher Scientific, catalog no. EP0741), 0.4 mM dNTPs (Invitrogen, catalog no. 18427088), 300 ng random primers (Invitrogen, catalog no. 48190-011), 10 U RiboLock RNase Inhibitor (Thermo Fisher Scientific, catalog no. EO0381) and water to 20 μL total volume. The sample was incubated at 50 °C for 1 h followed by 10 min at 75 °C.

*qPCR reactions and cycling conditions:*

Reactions were prepared with 5 μL of cDNA, 10 μL of TaqMan™ Universal PCR Master Mix (Thermo Fisher Scientific, catalog no. 4304437), 500 nM of each RRV-specific primer, and 250 nM of the corresponding probe or 900 nM of each 18S rDNA-specific primer, and 250 nM of the corresponding probe in a final volume of 20 μL. Amplifications were carried out in a CFX96 Touch real-time PCR detection system (Bio-Rad, Hercules, CA, USA).


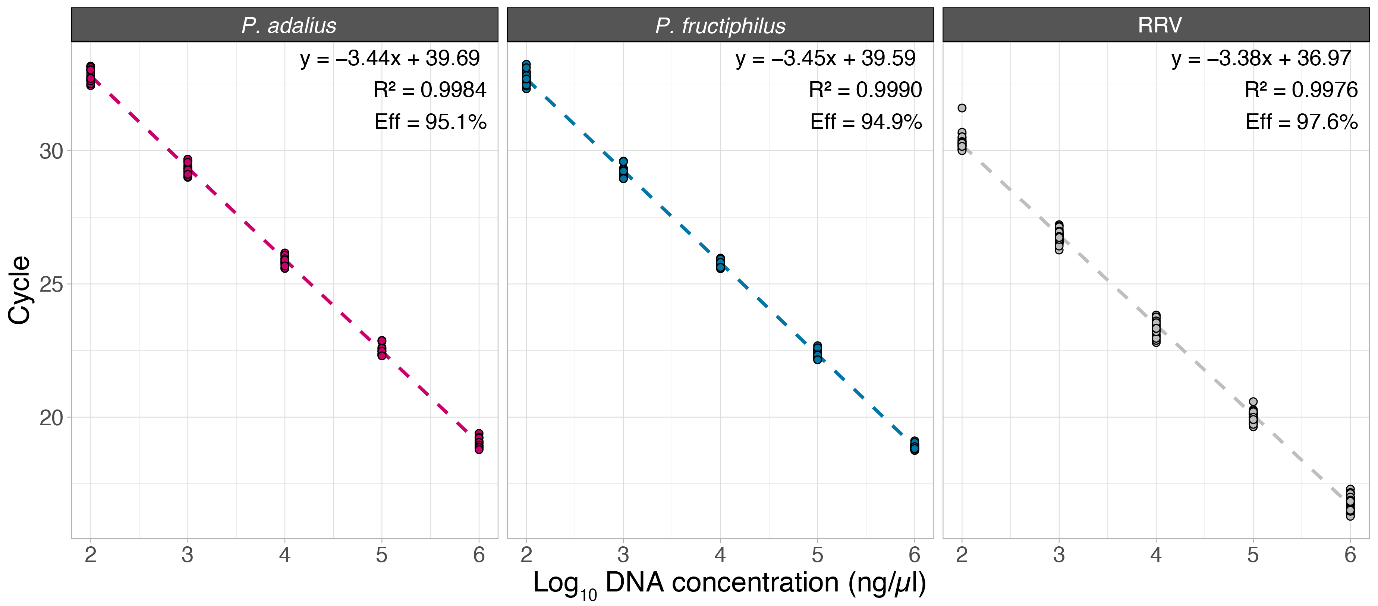


**Supplementary figure 1.** TaqMan™ qPCR standard curves generated using ten-fold dilutions of cDNA/DNA for multiple independent runs. (A) host (mite rDNA): *Phyllocoptes adalius,* (B) *P. fructiphilus* (B) and (C) Rose rosette emaravirus (RRV) load (cDNA). Standards were evaluated in independent assays to assess variation across plates.


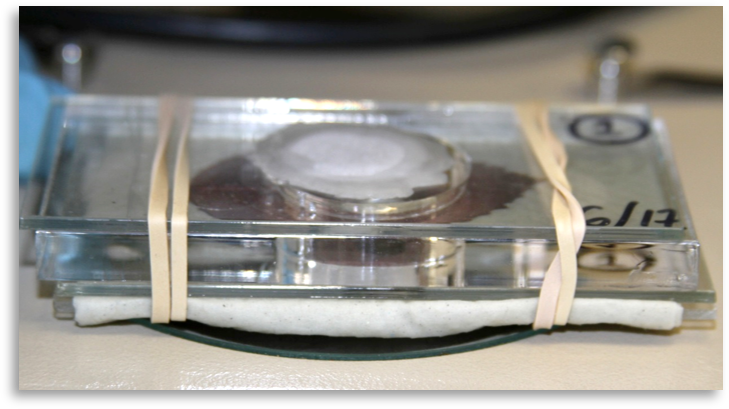


**Supplementary figure 2.** The modified Munger cell used for mite studies (Druciarek et al. 2024). The cell comprises layered Plexiglas pieces with a central cavity containing the mite specimens on a RRV-infected or virus-free leaflet placed on top of a moistened tissue paper. The setup is secured with rubber bands to maintain environmental stability and prevent mite escape during the observation period.
